# Changes in mitochondrial function and mitochondria associated protein expression in response to 2-weeks of high intensity interval training

**DOI:** 10.3389/fphys.2015.00051

**Published:** 2015-02-24

**Authors:** Grace Vincent, Séverine Lamon, Nicholas Gant, Peter J. Vincent, Julia R. MacDonald, James F. Markworth, Johann A. Edge, Anthony J. R. Hickey

**Affiliations:** ^1^Centre for Physical Activity and Nutrition Research, School of Exercise and Nutrition Sciences, Deakin UniversityMelbourne VIC, Australia; ^2^Department of Sport and Exercise Science, The University of AucklandAuckland, New Zealand; ^3^Department of General Practice and Primary Healthcare, Auckland School of Medicine, The University of AucklandAuckland, New Zealand; ^4^Applied Surgery and Metabolism Laboratory, School of Biological Sciences, The University of AucklandAuckland, New Zealand; ^5^Liggins Institute, The University of AucklandAuckland, New Zealand

**Keywords:** HIT, mitochondria, oxidative phosphorylation, PGC-1α, skeletal muscle

## Abstract

**Purpose:** High-intensity short-duration interval training (HIT) stimulates functional and metabolic adaptation in skeletal muscle, but the influence of HIT on mitochondrial function remains poorly studied in humans. Mitochondrial metabolism as well as mitochondrial-associated protein expression were tested in untrained participants performing HIT over a 2-week period.

**Methods:** Eight males performed a single-leg cycling protocol (12 × 1 min intervals at 120% peak power output, 90 s recovery, 4 days/week). Muscle biopsies (*vastus lateralis*) were taken pre- and post-HIT. Mitochondrial respiration in permeabilized fibers, citrate synthase (CS) activity and protein expression of peroxisome proliferator-activated receptor gamma coactivator (PGC-1α) and respiratory complex components were measured.

**Results:** HIT training improved peak power and time to fatigue. Increases in absolute oxidative phosphorylation (OXPHOS) capacities and CS activity were observed, but not in the ratio of CCO to the electron transport system (CCO/ETS), the respiratory control ratios (RCR-1 and RCR-2) or mitochondrial-associated protein expression. Specific increases in OXPHOS flux were not apparent after normalization to CS, indicating that gross changes mainly resulted from increased mitochondrial mass.

**Conclusion:** Over only 2 weeks HIT significantly increased mitochondrial function in skeletal muscle independently of detectable changes in mitochondrial-associated and mitogenic protein expression.

## Introduction

High-intensity, short-duration interval training (HIT) rapidly stimulates metabolic adaptations in skeletal muscle and improves aerobic capacity (Parra et al., 2000; Burgomaster et al., 2005, 2006, 2007, 2008; Gibala et al., 2006a, 2012; Gibala and McGee, 2008; Babraj et al., 2009). HIT is more effective than moderate-intensity continuous training for increasing aerobic power and managing risk factors related to the metabolic syndrome (Tjonna et al., 2008). So far, the majority of HIT research has used highly demanding training protocols typically including repetitive “all out” maximal sprinting efforts of ~30 s (Burgomaster et al., 2005; Gibala et al., 2006a). However, intense anaerobic exercise requires considerable participant motivation, which likely impacts its general prescription. Recently, more practical HIT models have been introduced, involving bouts of extended duration at a lower intensity interspersed with appropriate recovery periods (Little et al., 2011; Jacobs et al., 2013b). This modified form of HIT improves aerobic capacity and results in concurrent changes in mitochondrial associated mRNA and protein levels as well as in mitochondrial enzyme activity, indicating an enhanced oxidative potential (Hood et al., 2011; Little et al., 2011). Improvements in markers of metabolic control and arterial compliance are comparable to traditional endurance exercise training (Burgomaster et al., 2008; Rakobowchuk et al., 2008) and HIT is perceived to be more enjoyable (Bartlett et al., 2011). Therefore, HIT likely represents a time-efficient strategy to enhance whole body physiological function and prevent metabolic disease. However, few studies have explored the metabolic adaptations through which these improvements in exercise performance occur (Jacobs et al., 2013b).

Traditional endurance exercise protocols promote mitochondrial content (Holloszy, 1967; Bruce et al., 2004) and mitochondrial biogenesis (Wright et al., 2007a). Molecular signals such as Ca^+2^, 5′ adenosine monophosphate (AMP) and reactive oxygen species (ROS) increase post-exercise leading to the initial activation of mitochondrial biogenesis (McConell et al., 1997; Baar et al., 2003; Irrcher et al., 2009). The underlying exercise-induced mechanisms following cumulative exercise sessions have been well documented (Wright et al., 2007a), resulting in enhanced levels of peroxisome proliferator-activated receptor gamma coactivator (PGC-1α) gene expression. PCG-1α, a key regulator of mitochondrial biogenesis and function that positively regulates the mitochondrial network (Wu et al., 1999; Finck and Kelly, 2006; Handschin and Spiegelman, 2006), is upregulated in human skeletal muscle following acute endurance exercise (Cartoni et al., 2005; Russell et al., 2005) and low-volume HIT (Gibala et al., 2009; Little et al., 2010).

Short duration HIT has been shown to improve aerobic capacity (Burgomaster et al., 2005) and elevate mitochondrial enzyme activities (Gibala et al., 2006c; Hood et al., 2011; Little et al., 2011). Enzyme activity assays and mitochondrial-associated gene and protein expression provide static, surrogate measures of mitochondrial content and oxidative capacities. However, changes in these markers do not always reflect the collective function or the complexity of mitochondrial function (Larsen et al., 2012; Jacobs et al., 2013a). Indeed, they do not provide a measure of oxygen flux capacities (e.g., of the oxidative phosphorylation system (OXPHOS), and maximal uncoupled respiration in permeabilized fibers) or phosphorylation coupling efficiencies (Picard et al., 2011). In addition, alterations in mitochondrial function can occur independently from changes in mitochondrial associated protein expression (Viganò et al., 2008; Jacobs et al., 2013a). The permeabilized fiber method allows measurement of mitochondrial respiration *in situ* from as little as 2.5 mg of muscle (Pesta and Gnaiger, 2012), and provides a reference to the maximal oxidative power *in vivo* (Boushel et al., 2011), as well as an estimate of energy transfer efficiency through the degree of mitochondrial respiratory control. Furthermore, *in situ* analysis minimizes mitochondrial disruption that inevitably occurs during the isolation process (Daussin et al., 2008; Picard et al., 2011).

To our knowledge, only one previous study investigated mitochondrial function following a short period of HIT (Jacobs et al., 2013b). In addition, discrepancies in mitochondrial function and protein expression are not without precedent in the literature (Viganò et al., 2008; Jacobs et al., 2013a). However, mitochondrial function and mitochondrial associated protein expression have never been investigated in parallel following a HIT regime. Therefore, the aim of the present study was to investigate the changes in respiratory fluxes that underlie exercise performance responses to HIT using the permeabilized fiber method, as well as the expression levels of proteins associated with mitochondrial function. We hypothesized that 8 sessions of HIT over 2 weeks would increase markers of mitochondrial function and mitochondrial content within skeletal muscle.

## Materials and methods

### Participants

Eight male, moderately active participants (Mean ± SD, age 22 ± 2 years, body mass 81 ± 6 kg, height 1.82 ± 0.1 m) volunteered to participate in the study. Participants were screened for contraindications to exercise and gave written consent before participating. The study design and experimental procedures were approved by The University of Auckland Human Participants Ethics Committee (Ref. 2009/397).

### Familiarization and baseline exercise testing

This study was originally part of a larger trial aimed at looking at cross-education between limbs (Howatson et al., 2013; Magnus et al., 2013; Pearce et al., 2013). The foot of the contralateral leg was firmly strapped to a platform that replaced the crank arm on the non-exercising side. As we did not observe any exercise-induced change in any of the measured parameters, we decided to focus on the exercising leg only. Therefore, one-legged cycling was selected as the training modality. A preliminary session was conducted to familiarize participants with the one-legged cycling exercise mode. This comprised one 10 min bout of cycling at an intensity of 100 W for each leg, performed unilaterally. Forty eight hours post-familiarization participants performed two one-legged cycling graded exercise tests to volitional fatigue on an electromagnetically braked cycle ergometer (Schiller CH-6340, BAAR, Switzerland) to determine baseline peak power, time to fatigue, and VO_2_ peak.

The VO_2_ peak test commenced at a workload of 65 W with step-wise increments of 15 W every 2 min until cadence fell below 60 rpm. An open-circuit gas analysis system (Moxus modular oxygen uptake system, AEI technologies, Pittsburgh, PA, USA) was used to analyze expired air and determine peak oxygen consumption. The gas analysis system was calibrated prior to each test. Five days after the preliminary session, bilateral resting muscle biopsy samples were collected from *vastus lateralis*.

### Training protocol

Each participant was assigned a leg for HIT that was randomized by limb dominance in a counterbalanced fashion. The trained leg performed 12 × 60s intervals at 120% of peak power (as determined by the preliminary VO_2_ peak test) with a 90 s rest between intervals on the cycle ergometer. Sessions were performed 4 days per week for 2 weeks (8 sessions in total). A researcher measured recovery intervals and provided encouragement to facilitate maximal effort for each high-intensity interval. As in the exercise capacity tests, participants were required to remain seated during the training period. Participants refrained from participating in additional strenuous aerobic exercise or strength training outside of the prescribed training sessions during the study period. All participants completed all training sessions.

### Post-intervention tests

Approximately 48 h after completion of the last training session, a muscle biopsy sample was taken from each leg following an overnight fast. A further 48 h elapsed before participants performed two unilateral one-legged graded exercise tests to determine changes in peak power, time to fatigue, and VO_2_ peak.

### Skeletal muscle sampling and fiber preparation

Local anesthetic (2% Xylocaine) was injected into the skin overlying the *vastus lateralis* and a small incision made to the skin and underlying tissue. A 5 mm Bergstrom needle was then inserted into the belly of the *vastus lateralis* muscle. Manual suction was applied and the biopsy sample excised. Extracted tissue was then divided into two ~50 mg samples, and a sample for mitochondrial respiration analysis was washed with saline solution and placed into an 1.7 ml micro-centrifuge tube containing ice-cold high energy relaxing solution (BIOPS, from here on in mmol·L^−1^ unless stated 10 Ca-EGTA buffer, 0.1 free calcium, 20 imidazole, 20 taurine, 50 K-MES (potassium 2-(*N*-morpholino)ethanesulfonic acid), 0.5 dithiothreitol, 6.56 MgCl_2_, 5.77 ATP, 15 phosphocreatine, at pH 7.1. The remaining sample was frozen immediately in liquid nitrogen and stored in a −80°C freezer for later analysis.

BIOPS muscle stored was teased into small fiber bundles within 2 h of collection, as previously described (Pesta and Gnaiger, 2012). Muscle fiber bundles were then placed into 1 ml ice-cold BIOPS and freshly prepared saponin (50 μg) was added to permeabilize the plasma membrane. Fibers were then gently shaken in culture plates for 30 min at 4°C. Fibers were rinsed 3 times for 10 min in 1 ml ice-cold incubation MiRO5 assay medium (0.5 EGTA, 3 MgCl_2_, 60 K-lactobionate, 20 taurine, 10 KH_2_PO_4_, 110 sucrose, and 1 mg·ml^−1^ BSA in 20 HEPES, pH 7.1 at 30°C) to remove the saponin, cytosol, and adenine nucleotides. Fiber bundles were blotted dry using filter paper and 5 mg of skeletal muscle tissue weighed for respiration assays.

### Mitochondrial respiration

Mitochondrial respiration in permeabilized muscle fibers was analyzed using two OROBOROS® O2K oxygraphs (Anton Paar, Graz, Austria) with chambers adjusted to 2 ml at 30°C. Respiration was measured as weight-specific oxygen flux (pmolO_2_·s^−1^·mg^−1^) wet weight, calculated as the time derivative of oxygen concentration using the DatLab 4 Analysis Software, OROBOROS® (Oroboros Instruments, Innsbruck, Austria).

The assay protocol consisted of the sequential titration of various substrates, inhibitors, and uncoupling agents of the respiratory chain to the skeletal muscle in MiRO5 containing catalase (1558 U·ml^−1^). Fibers were super-saturated with oxygen by injecting oxygen into the header space above samples after calibration and levels maintained between 280 and 450 nmol·ml^−1^. All assays were performed in duplicate.

We chose a substrate/inhibitor titration protocol targeting fluxes through aerobic glycolytic routes. Basal oxidation was first measured, then 10 mM glutamate and 2 mM malate were added and the LEAK state measured (GM leak). The addition of excess 1.25 mM ADP tested Complex I (CI) dependent oxidative phosphorylation with GM (GM-ADP), and the addition of cytochrome-*c* (10 μM) tested the functional integrity of mitochondrial outer membranes. The additional effect of pyruvate (10 mM) was used to maximize CI mediated flux (OXPHOS-CI) followed by the Complex II (CII) substrate succinate (10 mM) for combined Complex I and Complex II flux (OXPHOS-CI, CII), and this combination of substrates aimed to maximize mitochondrial O_2_ flux capacities.

Inhibition of the phosphorylation system by addition of atractyloside (0.25 mM) resulted in a transition to State 4 respiration (CI, CII Leak) by blocking the adenine nucleotide translocase (ANT). In excess ADP, like GDP inhibits uncoupling protein conductance (UCPs). As atractyloside also inhibits proton leak through ANT, any residual Leak respiration results from proton flux through the inner mitochondrial membrane.

**Figure 1:**
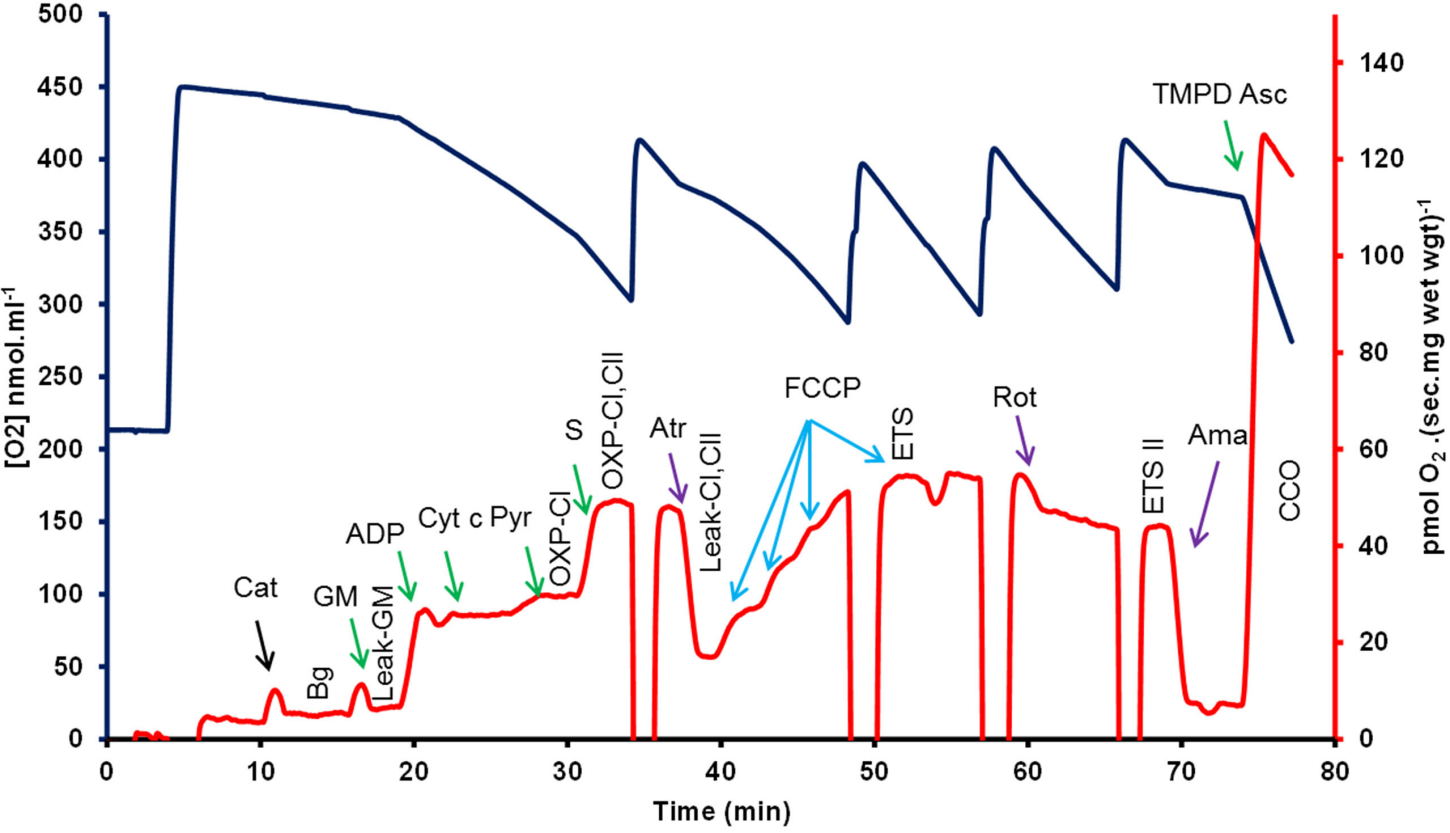
**A representative trace of the respiration substrate inhibitor uncoupler titration protocol used in this study to test saponin permeabilized *vastus lateralis* muscle (pre-HIT)**. The change in oxygen concentration (blue line) and the time derivative per unit mass (red line and axis) provided the flux of permeabilized *vastus lateralis* fibers over time following the addition of mitochondrial substrates (green), poisons (purple) and the uncoupling agent FCCP, carbonyl cyanide p-(trifluoromethoxy) phenyl-hydrazone (FCCP, blue). GM-Leak—glutamate and malate, ADP addition initiates oxidative phosphorylation (OXPHOS) with GM. Cyt-c—addition of cytochrome *c* tested mitochondrial integrity. OXPHOS-CI—GM-ADP plus pyruvate measured CI flux, and OXPHOS-CI, CII—represents the addition of succinate to maximize Complexes I and II flux in OXPHOS. CI, CII-Leak—Atractyloside (Atr) inhibits OXPHOS and forces leak respiration with CI and CII, while ETS-CI, CII—results from sequential titration of FCCP to uncouple the electron transport system from OXPHOS. Respiration states used in this study are indicated (vertical text bolded), Bg, background; Leak-GM, leak respiration with GM; OXPHOS-CI, oxidative phosphorylation supported by Complex I (CI) substrates; OXPHOS-CI, CII, OXPHOS supported by CI and Complex II (CII) substrates; ETS, electron transport system (ETS) flux supported by CI and CII; ETS-II, ETS supported by CII; and CCO, cytochrome c oxidase. ETS-CII—was measured by the addition of rotenone (Rot). Lastly CCO results from the inhibition of Complex III with antimycin-a (Ama), followed by the addition of TMPD-Asc, N, N, N′, N′-tetramethyl-p-phenylendiamine + ascorbate; Cat, catalase (concentrations are outlined in the methods); GM, glutamate + malate; ADP, adenosine diphosphate; cyt-c, cytochrome-c; Succ, succinate; atr, atrylactoside; rot, rotenone; Ama, Antimycin A.

The electron transport system (ETS) was then uncoupled from the inhibited phosphorylation system by repeated titrations of 1 μL of carbonyl cyanide p-(trifluoromethoxy) phenyl-hydrazone (FCCP, 0.5 μM). Under these conditions this state represents the maximal rate of the ETS (ETS CI, CII). Complex I (ETS CII) and then Complex II and III activity were selectively and sequentially inhibited by the addition of rotenone (1 μM), 15 mM malonate and antimycin-*a* (1 μM), which act to block the flow of electrons from Complexes I, II, and III respectively. Finally, the activity of CCO was measured by addition of the electron donor couple N, N, N′, N′-tetramethyl-p-phenylendiamine (TMPD, 0.5 mM) and ascorbate (2 mM). Results were then corrected for background chemical O_2_ consumption and also normalized to citrate synthase (CS) activity that were measured in matched samples. Control ratios were determined as follows; The RCR-1 = the ratio of oxidative phosphorylation (OXPHOS) derived oxygen flux relative to leak respiration flux supported by complex I (CI) substrates. RCR-2 = the ratio of OXPHOS and leak respiration fluxes supported by CI and Complex II. % CI/ETS = percentage contribution of CI to the chemically uncoupled ETS oxygen flux. CCO/ETS = the ratio of cytochrome *c* oxidase (CCO) relative to the ETS (CCO/ETS) provided a measure of the flux through CCO relative to the ETS. A representative trace of the respiration substrate, inhibitor and uncoupler titration protocol is shown in Figure 1.

### Citrate synthase assay

Stored tissue was equilibrated with 1:40 volumes (w:v) ice-cold homogenization buffer (25 Tris-HCl pH 7.8, 1 EDTA, 2 MgCl_2_, 50 KCl, 0.5%v/v Triton X-100). Samples were then homogenized and citrate synthase (CS) activity determined using a plate reader (Molecular Devices Spectramax 340, Sunnyvale, USA) and 5 μl of sample supernatant was added to an assay media containing Tris-HCl (50 mM, pH 8.0), acetyl coenzyme A (0.1 mM), DTNB (0.2 mM). Reactions started by addition of 5 mM oxaloacetate (Sigma, St. Louis, USA), and followed the formation of mercaptide ions at 412 nm. CS activity (units per wet weight of tissue) was determined relative to CS standard (Sigma, St. Louis, USA) from porcine heart. CS data were used as a proxy of mitochondrial mass and used to normalize flux rates in order to estimate mitochondrial specific fluxes.

### Protein extraction and immunoblotting

Frozen tissue (20–30 mg) was mechanically disrupted in 15 μL ice cold lysis buffer/mg (in mM, 20 Tris-HCL (pH 7.4), 137 NaCl, 1% NP-40, 10% glycerol, 1 EDTA, with Halt Protease, and Phosphatase Inhibitor Cocktail (Thermo Scientific Pierce) with 2.8 mm ceramic beads using the Omni Bead Ruptor Homogenizer bead mill (Omni International) at 5.65 m·s^−1^, 2 × 30 s. Homogenates were then rotated for 1 h at 4°C then centrifuged at 13,000 *g* for 15 min at 4°C and supernatants stored at −80°C. Protein contents were determined by the bicinchoninic acid method (Thermo Scientific Pierce). Samples were diluted to 20 μg protein in 4× Laemmli loading buffer, boiled for 5 min, and separated by SDS-polyacrylamide gel electrophoresis (SDS-PAGE). Protein was transferred to PVDF membranes using a semi-dry Trans-Blot® Turbo™ Transfer System (Biorad) with ready-to-use Trans-Blot® Turbo™ Mini PVDF Transfer Packs (Biorad). Membranes were blocked in 5% BSA/Tris Buffer Saline/0.1% Tween 20 (TBST) for 2 h at room temperature, followed by incubation overnight with gentle agitation at 4°C with an anti-PGC-1 Antibody (Millipore, AB3242) or an optimized premixed cocktail antibody (Abcam, ab110411, MitoProfile® Total OXPHOS Human) for oxidative phosphorylation proteins (Abcam, Science Park Cambridge, UK) targeting CI subunit NDUFB8 (ab110242/MS105), CII subunit 30 kDa (ab14714/MS203), Complex III subunit Core 2 (ab14745/MS304), Complex IV subunit (CCO) II (ab110258/MS405), and ATP synthase subunit alpha (ab14748/MS507). Membranes were washed for 30 min with TBST and probed with goat anti-rabbit (for PGC-1α) or anti-mouse (for MitoProfile) IgG peroxidase conjugated secondary antibodies (Thermo Scientific Pierce) for 1 h at room temperature. Membranes were washed for 30 min in TBST and protein bands were visualized using ECL Prime Western Blotting Detection Reagent (GE healthcare Amersham), and signals quantified using ImageQuant LAS 4000 (FUJI film). Densitometry analysis employed Kodak Molecular Imaging Software (Version 4.0.5, © 1994-2005 Eastman Kodak Company). Equal protein loading was verified by stripping and reprobing membranes with anti-GAPDH (6C5) (Mouse Monoclonal IgG1, Abcam: ab8245).

### Statistical analyses

Paired sampled *t*-tests were used to test between pre- and post-training measures. Diagnostic plots of residuals and fitted values were checked to ensure homogeneity of variance (a key assumption for *t*-tests). Consequently, all data were log10-transformed and analyses were conducted on these transformed scales. Statistical significance was set at *p* ≤ 0.05 and all data are presented as mean ± SEM unless otherwise stated. Note that the statistical significance reported in the figures is based on analysis of the transformed data but the reported means ± SEM are on the original (untransformed) scale.

## Results

### Performance measures

Following 2 weeks of HIT, peak power and time to fatigue increased by 22 and 33%, respectively (*P <* 0.01). There was no significant increase in relative VO_2_ peak following training. All performance data are reported in Table 1.

**Table 1:** **Performance and mitochondrial data pre- and post-HIT**.

	**Pre-HIT**	**Post-HIT**	***P*-value**
**A. PERFORMANCE**			
VO_2_ peak (ml·kg^−1^·min^−1^)	45.7 ± 2.1	50.8 ± 1.0	0.124
Peak power (W)	151.2 ± 6.4	184.5 ± 9.1	0.002
Time to fatigue (s)	809.3 ± 51.3	1075.7 ± 72.8	0.002
**B. MITOCHONDRIAL**			
RCR-1	11.2 ± 1.3	16.3 ± 3.4	0.459
RCR-2	3.5 ± 0.1	4.1 ± 0.3	0.161
% CI of ETS	47.4 ± 3.3	50.9 ± 2.4	0.541
CCO/ETS	2.0 ± 0.1	2.2 ± 0.1	0.120
Citrate synthase (CS) (μmol·min^−1^·mg^−1^ wet wgt)	0.50 ± 0.1	0.88 ± 0.1	0.038

*Effects of HIT on performance measures, control ratios, fluxes and citrate synthase activity pre- and post-HIT. The RCR-1 represents the ratio of oxidative phosphorylation (OXPHOS) derived oxygen flux relative to leak respiration supported by complex I (CI) substrates (see Figure 1). The RCR-2 represents the ratio of OXPHOS and leak respiration fluxes supported by CI and Complex II substrates. The addition of rotenone permitted an estimate of the percentage contribution of CI to the chemically uncoupled electron transport system oxygen flux (ETS). The ratio of cytochrome c oxidase (CCO) relative to the ETS (CCO/ETS) provided a measure of the flux through CCO relative to the ETS. Data are presented as mean ± SEM (n = 8)*.

### Citrate synthase activity

In post-HIT samples, CS activity increased by 43% (*P* < 0.05, Table 1) when compared to baseline pre-training levels.

### Mitochondrial function

**Figure 2:**
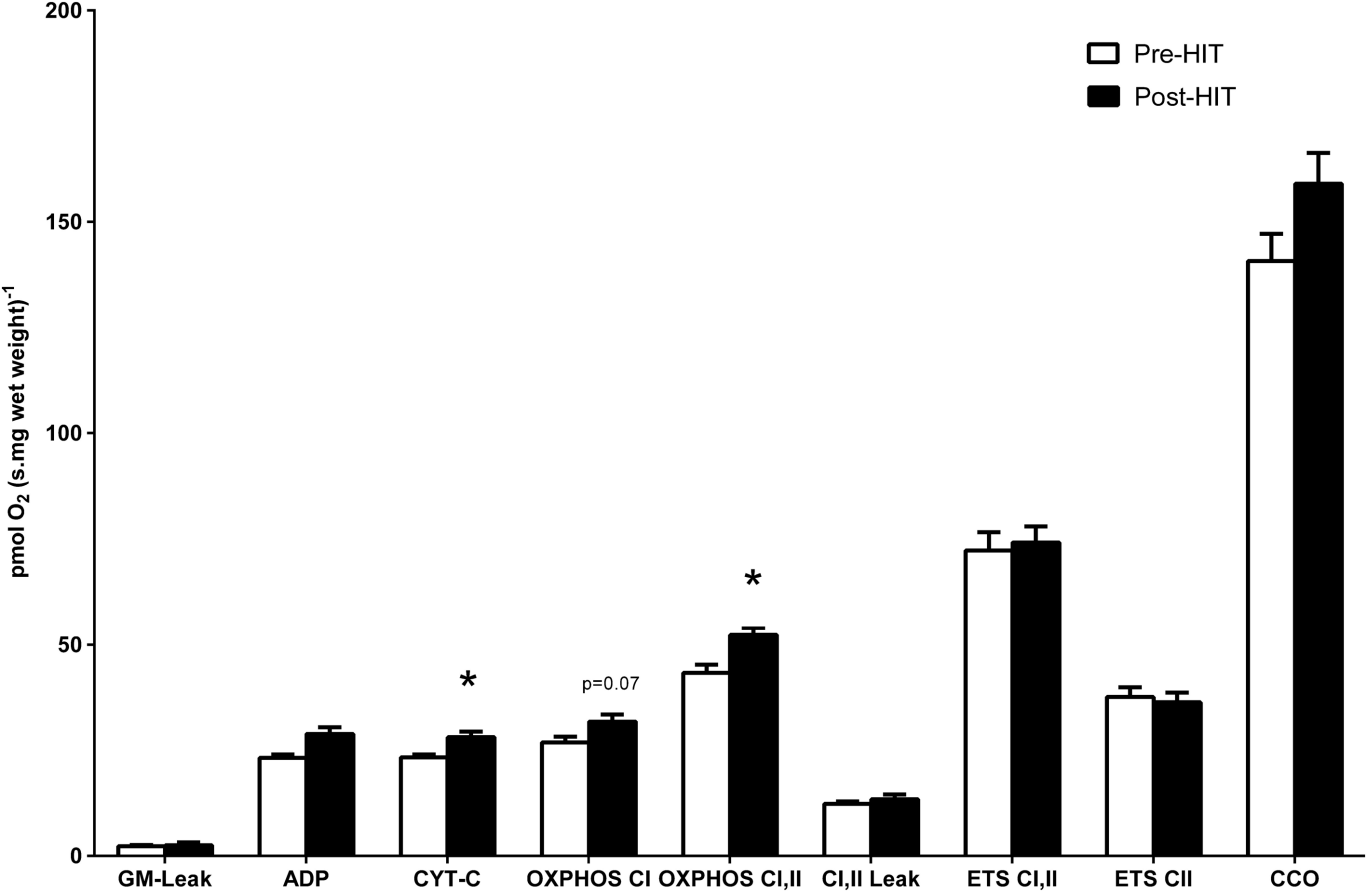
**In response to HIT, respiratory flux showed elevation of CYT-C and OXPHOS CI, II**. ^*^Denotes significance (*P* < 0.05), error bars represent SEM, *n* = 8. Note that the statistical significance reported in the figures is based on analysis of the transformed data but the reported means ± SEM are on the original (untransformed) scale.

A representative trace of the respiration substrate, inhibitor and uncoupler titration protocol is shown in Figure 1. Respiratory fluxes in response to HIT are shown in Figure 2. The addition of exogenous cytochrome-c provided no evidence of compromised mitochondrial membrane integrity. The corresponding respiratory flux (CYT-C) increased by 21% post-HIT when compared to baseline pre-HIT levels (*P* < 0.05).

The effect of pyruvate (Pyr) was used to maximize CI mediated flux (OXPHOS-CI), followed by the addition of the Complex II (CII) substrate succinate (S) to maximize combined CI and CII flux (OXPHOS-CI, CII). While the respiratory flux for OXPHOS CI increased with HIT, this increase did not reach statistical significance (*P* = 0.07). Addition of succinate led to a 21% increase in respiratory flux (OXPHOS CI, II) in permeabilized fibers following HIT (*P* < 0.001). However, when respiration rates were expressed per unit of CS, no significant differences were apparent in CYT-C or OXPHOS-CI, II (data not shown).

Following this, the phosphorylation system was inhibited by addition of atractyloside (Atr) and the electron transport system (ETS) was then uncoupled from the inhibited phosphorylation system by FCCP titrations. Finally, CI (ETS CII), CII and III activity were selectively and sequentially inhibited by the addition of rotenone (Rot), malonate and antimycin-a (AMA). Lastly, the activity of CCO was measured by addition of the electron donor couple N, N, N′, N′-tetramethyl-p-phenylendiamine (TMPD) and ascorbate. No difference was noted between pre- and post-training for any of these respiration fluxes. RCR-1, RCR-2, % CI of ETS and COO/ETS remained unchanged following training (Table 1).

### Immunoblot analyses

**Figure 3:**
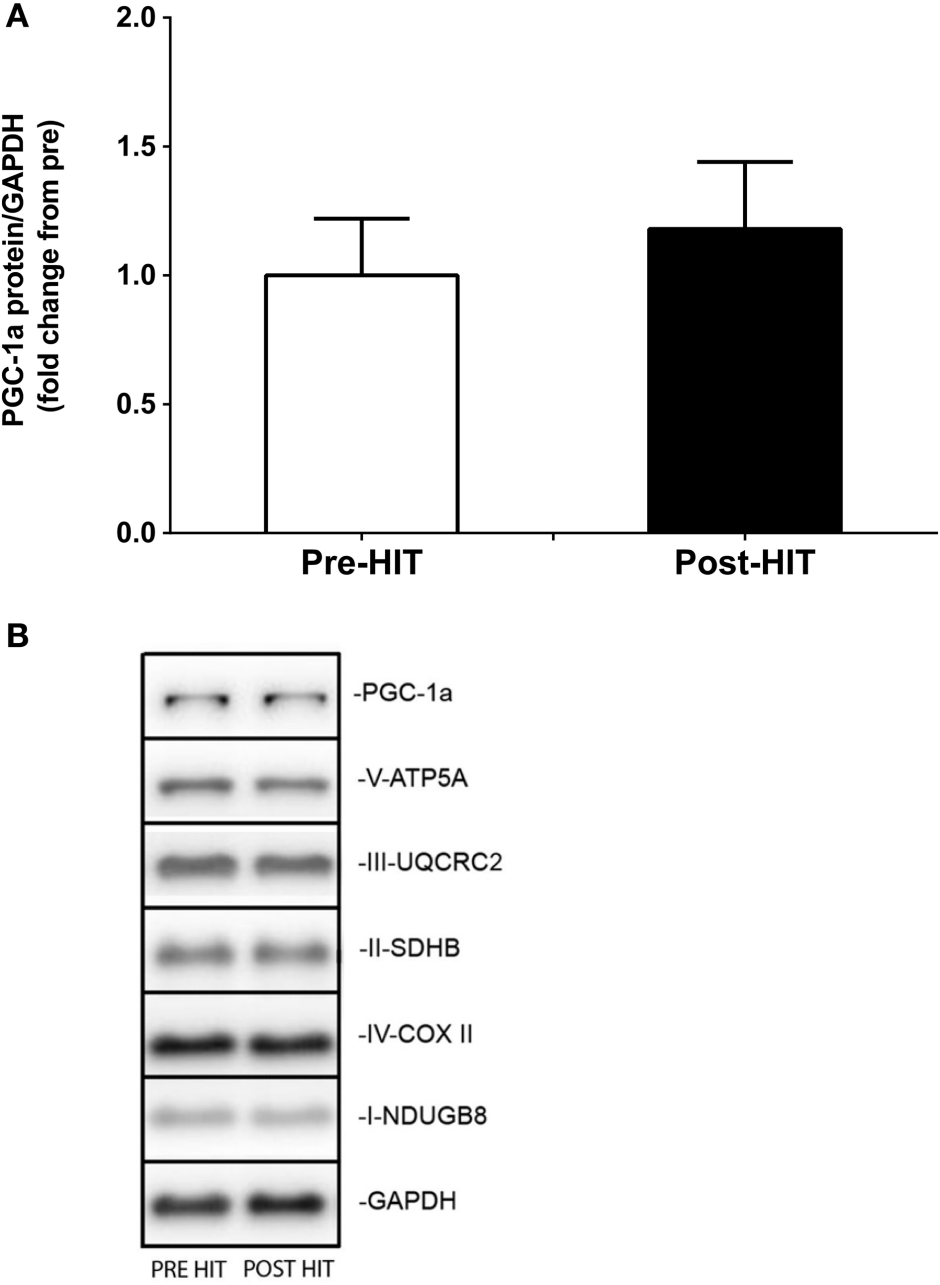
**Immunoblot analysis of the PGC-1α protein expression in response to HIT**. Mean ± SEM PGC-1α protein expression (*n* = 8) pre- and post-training. Note that the statistical significance reported in the figures is based on analysis of the transformed data but the reported means ± SEM are on the original (untransformed) scale. **(A)** Representative western blot images of the following proteins: PGC-1α, V-ATP5A, III-UQCRC2, II-SDHB, IV-COX II, I-NDUGB8, GAPDH whole muscle homogenates, pre- and post-HIT for one subject **(B)**.

Immunoblot blot analysis of PGC-1α protein expression showed no significant effect of HIT (Figure 3A). As such, representative blots showing PGC-1α are displayed (Figure 3B). Similarly, none of the mitochondrial specific markers of OXPHOS or ETS measured (PGC-1α, V-ATP5A, III-UQCRC2, II-SDHB, IV-COX II, I-NDUGB8, GAPDH) could resolve differences due to HIT (Figure 3B).

## Discussion

This study is the first to simultaneously evaluate the effects of HIT on mitochondrial respiration and expression of mitochondrial-associated proteins. We showed that only 2 weeks of low-volume HIT improved exercise performance and increased mitochondrial respiration flux capacities. However, we detected no increases in protein expression of PGC-1α or OXPHOS or ETS mitochondrial markers. Increased leg muscle power was mirrored by elevated OXPHOS supported combined complex I and II electron inputs (OXPHOS-CI, CII), and this increase matched increases in citrate synthase enzyme activities, a marker of mitochondrial content (Larsen et al., 2012). Our findings indicate that low-volume HIT improves muscle oxidative capacity, mostly through an increase in mitochondrial mass/volume.

The respective improvements of 18 and 25% for peak power and time to fatigue observed in this study are comparable to previous work involving similar training protocols (Little et al., 2011) or overall training intensities and durations (Rodas et al., 2000). While no significant increase in VO_2_ peak following 2 weeks of HIT has been reported before (Burgomaster et al., 2005, 2006), minor increases have also been observed (Jacobs et al., 2013b). HIT protocols of a longer duration (6 weeks) do however increase VO_2_ peak to the same extent as traditional endurance training (Burgomaster et al., 2008).

HIT significantly increased maximal OXPHOS flux supported by Complex I and Complex II substrates (OXPHOS-CI, CII), while a trend was apparent for OXPHOS supported by Complex I (CI) substrates only. Others have reported improvements in *in vivo* oxidative capacity following six sessions of HIT using phosphorous magnetic resonance spectroscopy (Forbes et al., 2008; Larsen et al., 2013). Maximal OXPHOS with CI and CII is consistent with others findings employing the permeabilized fiber method with a short HIT protocol (Jacobs et al., 2013b). Jacobs et al. (2013b) also reported an increase in OXPHOS and ETS flux with multiple electron inputs (including lipids via electron transferring flavoprotein, ETF), but not with just the complex II substrate succinate. While the lack of significance in ETS states may reflect the relatively small sample size of our study (*n* = 8) and/or the lower overall training duration across the 2 weeks period, the activation of both CI and CII appears to better reveal muscle mitochondrial adaptation to HIT.

Compared to pre-HIT values, we observed a 43% increase in CS activity, an elevation that is greater than in other studies with similar effort and duration (Rodas et al., 2000; Little et al., 2010). CS activity is a quantitative indicator of oxidative capacity and is used to normalize global measures of muscle bioenergetic capacity (Larsen et al., 2012). On normalizing OXPHOS-CI, CII capacity to citrate synthase activities no differences were apparent between pre- and post-training respiratory states. We also report no significant improvement in the RCR-1, RCR-2, % CI ETS, and the ratio of cytochrome *c* oxidase (CCO) relative to the ETS (CCO/ETS) in muscle fibers. Increases in RCRs theoretically indicate greater ATP yields per oxygen molecule and therefore greater OXPHOS efficiency (Pesta and Gnaiger, 2012), as reported in active/athletic vs. sedentary individuals (Zoll et al., 2004; Coen et al., 2013). Similarly, changes in CCO/ETS indicate alterations in pathway flux control and efficiencies (Pesta and Gnaiger, 2012). Our results suggest that alterations to muscle oxidative capacity are mostly enhanced through quantitative rather than qualitative changes in mitochondria. Still an explanation is required for the more substantive changes in time to fatigue and peak power output relative to the lesser increases in mitochondrial flux (~10–15%). Mitochondria need not operate at full capacity, as respiration of permeabilised *vastus lateralis* muscle fibres is greater in vitro than the apparent respiration rates of thigh muscle measured *in vivo*. However, these improvements may also result from increased non-bicarbonate muscle pH buffering capacities (Edge et al., 2006; Gibala et al., 2006b), a parameter not measured in this study.

In contrast to changes in mitochondrial function, there was no change in the protein expression of PGC-1α or of mitochondrial markers of OXPHOS or ETS. Endurance (Russell et al., 2003) and HIT protocols (Gibala et al., 2009) have reported increases in PGC-1α mRNA and protein contents. However, discrepancies between alterations in the mitochondrial function and associated changes in protein expression have previously been noted (Viganò et al., 2008; Jacobs et al., 2013a). Total PGC-1α protein content may not reflect PCG-1α activation (Wright et al., 2007b). On activation PGC-1α translocates to the nucleus (Rim et al., 2004; Cowell et al., 2007; Sano et al., 2007; Wright et al., 2007b) and does so in stimulation by HIT (Little et al., 2010). Translocation was not testable in muscle homogenates in our study. However, prolonged (6 weeks) HIT increased the whole muscle protein content of PGC-1α by 100% in young healthy individuals (Burgomaster et al., 2008). Thus, mitochondrial capacity appears to improve through expansion prior to measurable changes in PGC-1α expression with limited exposure to HIT.

HIT also did not induce measurable changes in the protein expression levels of the respiratory complex components CI subunit NDUFB8, CII subunit 30 kDa, CIII subunit Core 2, CIV subunit (CCO) II and ATP synthase subunit alpha. CS is a validated marker of mitochondrial biogenesis (Larsen et al., 2012). However, it was also suggested that complex II and complex IV protein content are alternative suitable markers of mitochondrial content (Larsen et al., 2012). In the present study, despite an increase in CS activity, we observed no change in the levels of CII and CCO proteins. This may be indicative of intrinsic alterations of mitochondrial function. Qualitative changes in the OXPHOS process, or regulators of mitochondrial respirational flux (e.g., NOS and NO) might partly account for the observed increase in respiration rates. This may in turn explain the lack of change in the expression levels of respiratory complex components.

In conclusion, the current study demonstrates that skeletal muscle mitochondrial function, but not mitochondrial-associated protein expression, is modestly altered following only 2 weeks of HIT in young untrained males. It indicates that HIT provides a time-efficient training strategy for improving skeletal muscle mitochondrial function.

### Conflict of interest statement

The authors declare that the research was conducted in the absence of any commercial or financial relationships that could be construed as a potential conflict of interest.
